# Supplementary figures and images for: Genome Analysis of Anti-Phage Defense Systems and Defense Islands in Stenotrophomonas maltophilia: Preservation and Variability
Source: Viruses. 2024 Dec 10;16(12):1903. doi: 10.3390/v16121903 (PMC11680222; doi:10.3390/v16121903)

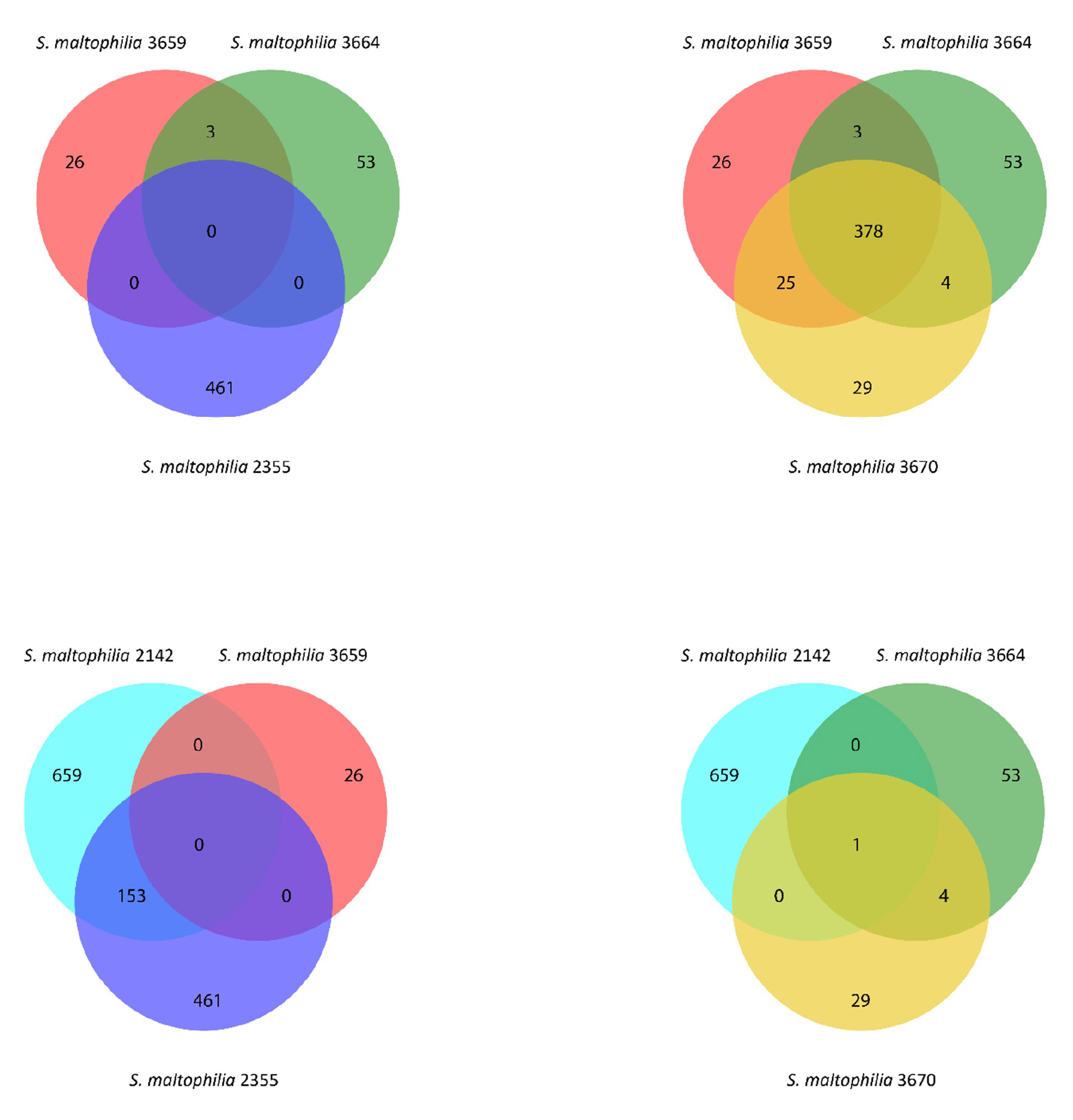

Supplement: Supplementary file 1 [file viruses-16-01903-s001.zip › Supplementary/Figure S1.jpg]
